# Performance-based tests versus behavioral ratings in the assessment of executive functioning in preschoolers: associations with ADHD symptoms and reading achievement

**DOI:** 10.3389/fpsyg.2015.00545

**Published:** 2015-04-29

**Authors:** Ana Miranda, Carla Colomer, Jessica Mercader, M. Inmaculada Fernández, M. Jesús Presentación

**Affiliations:** ^1^Departamento de Psicología Evolutiva y de la Educación, Universidad de ValenciaValencia, Spain; ^2^Departamento de Educación, Universidad Jaume I, CastellónSpain; ^3^Departamento de Psicología Evolutiva, Educativa, Social y Metodología, Universidad Jaume I, CastellónSpain

**Keywords:** executive functioning, performance-based tests, BRIEF, ADHD, reading, preschool

## Abstract

The early assessment of the executive processes using ecologically valid instruments is essential for identifying deficits and planning actions to deal with possible adverse consequences. The present study has two different objectives. The first objective is to analyze the relationship between preschoolers’ performance on tests of Working Memory and Inhibition and parents’ and teachers’ ratings of these executive functions (EFs) using the *Behavior Rating Inventory of Executive Function* (BRIEF). The second objective consists of studying the predictive value of the different EF measures (performance-based test and rating scales) on Inattention and Hyperactivity/Impulsivity behaviors and on indicators of word reading performance. The participants in the study were 209 children in the last year of preschool, their teachers and their families. Performance-based tests of Working Memory and Inhibition were administered, as well as word reading measures (accuracy and speed). The parents and teachers filled out rating scales of the EF and typical behaviors of attention deficit hyperactivity disorder (ADHD) symptomatology. Moderate correlation values were found between the different EF assessments procedures, although the results varied depending on the different domains. Metacognition Index from the BRIEF presented stronger correlations with verbal working memory tests than with inhibition tests. Both the rating scales and the performance-based tests were significant predictors of Inattention and Hyperactivity/Impulsivity behaviors and the reading achievement measures. However, the BRIEF explained a greater percentage of variance in the case of the ADHD symptomatology, while the performance-based tests explained reading achievement to a greater degree. The implications of the findings for research and clinical practice are discussed.

## Introduction

Executive function (EF) is a complex construct that encompasses a wide variety of processes, including planning, working memory, attention, inhibition, or mental flexibility, and basically depends on the prefrontal areas and the reticular-thalamic system (Goldstein et al., 2013). In spite of different nuances, the majority of the experts agree that, synthesizing, the term EF refers to skills that make it possible to regulate one’s own behavior and thinking processes in order to achieve a desired goal. Therefore, these skills play a decisive role in academic, emotional and social adaptation (Posner and Rothbart, 2000; Barkley, 2011).

The set of EFs evolve throughout the life cycle (Dawson and Guare, 2010), but the preschool years, an important preparation period for formal education, are an extremely sensitive phase in their development. Empirical studies have shown a qualitative leap in EF between 3 and 6 years-old, with a considerable increase in basic competences such as working memory, cognitive flexibility or inhibitory control of behavior (Garon et al., 2008; Zelazo et al., 2008; Montgomery and Koeltzow, 2010; Cartwright, 2012). The advances that occur are associated with an increase in basic reasoning skills (Richland and Burchinal, 2013) and with a qualitative change in the regulatory function of language (Jacques and Zelazo, 2005; Alarcón-Rubio et al., 2014). Consequently, research in the field of neuroscience has confirmed that during the preschool period there is a significant increase in the number of synapses in the prefrontal cortex, the part of the brain associated with attention, working memory and self-regulation (Twardosz, 2012). Furthermore, there are theoretical assumptions that mental set shifting in early childhood builds on working memory and inhibitory control processes (Diamond, 2013), which has been empirically demonstrated (Brocki and Tillman, 2014). Therefore, the present study has focused on the EFs of working memory and inhibition.

The early assessment of the complex spectrum of executive processes, therefore, is essential for promoting optimal development, identifying deficits and planning actions to deal with possible adverse consequences (Manchester et al., 2004; Diamond and Lee, 2011; Anderson and Reidy, 2012). There are currently two different approaches to assessing EF in preschool children. On the one hand, performance-based tests are designed to evaluate specific aspects, and they are administered under strict laboratory conditions. The ecological validity of these tests has been questioned, based on the argument that there could be a poor match between what the child has to do in the structured evaluation setting and the demands of real life. On the other hand, the other assessment approach, represented by the rating scales, involves an attempt to remedy this methodological limitation by proposing the objective of assessing these skills in natural contexts in order to gain ecological validity. In this case, in order to integrate multi-source data, parents and teachers provide information about day-to-day life outside the structured evaluation context. The pioneer work in this field was carried out by the Gioia group, who developed an EF rating scale called the *Behavior Rating Inventory of Executive Function* (BRIEF; Gioia et al., 2000; Isquith et al., 2004).

There are at least two core questions related to EF assessment in preschoolers that have hardly been studied in spite of their implications. The first refers to analyzing the agreement between performance-based EF tests and parents’ and teachers’ rating scales, and more specifically, the BRIEF. Studies carried out with children and adolescents have found moderate relationships (correlation coefficients between 0.01 and 0.48) between the two EF assessment methods in heterogeneous clinical samples (Anderson et al., 2002; Bodnar et al., 2007; Parrish et al., 2007). A exhaustive review published recently (Toplak et al., 2013) offers suggestive data on this topic. Thirteen of the 20 studies included in the review had used the BRIEF Inventory (eight with samples of children, four with adolescents and one with young adults). The 13 studies provided data on 182 correlations between the BRIEF and performance-based test, of which only 19% reached significant values, suggesting that performance-based and rating measures of EF assess different underlying mental constructs. As Toplak et al. (2013) conclude, one should not assume that performance-based and ratings measures of EF capture the same level of analysis or underlying process. Therefore these measures should not be used interchangeably as parallel measures of EF in clinical or educational assessments.

A second question pending study is related to the capacity shown by the two types of measures, performance-based EF tests and EF ratings, to predict validity criteria for functionality. Among these criteria, the attention deficit hyperactivity disorder (ADHD) justifiably stands out, as research has supported its interpretation as involving an essential EF deficit (Brown, 2013; Sánchez-Pérez and Gónzalez-Salinas, 2013). Another criterion with proven external validity is reading achievement, due to the demands of coordinating various cognitive skills that depend on an adequate EF (Cartwright, 2012).

Probably one of the most widely studied aspects in children with ADHD consists of EF deficits assessed with performance-based tests, as reflected in the review carried out by Willcutt et al. (2005). Even though they have been studied much less, these relationships have also been found during the preschool period, as shown in the meta-analysis carried out by Pauli-Pott and Becker (2011) on the strength of associations between EF in the preschool period and ADHD symptoms. This research found statistically significant weighted mean effect sizes in the different EF assessed. Specifically, the mean effect size for response inhibition was medium to large, while the mean effect size for working memory was small. Furthermore, the literature shows that preschoolers who scored high on ADHD-symptoms presented worse performance on working memory and inhibition tests than children with low levels of ADHD symptomatology rated by parents (Sonuga-Barke et al., 2003; Schoemaker et al., 2013) and teachers (Thorell and Wåhlstedt, 2006).

The meta-analysis conducted by Pauli-Pott and Becker (2011) also showed that performance-based EF test during the preschool period predicted not only concurrent but also school-age ADHD symptoms. In this sense, a longitudinal study (Brocki et al., 2010) carried out with a sample of preschoolers at risk of ADHD is especially relevant. The EF assessed with performance-based tests in the preschool period (at the age of 5) predicted ADHD symptomatology in the first years of the school-age period (at the age of 7). Specifically, both working memory and inhibition were related to inattention; at the same time, inhibition was also related to hyperactivity. Two basic ideas stem from these results: inhibition and working memory seem to be particularly strong correlates of ADHD symptoms in young children; and EF deficits in preschoolers maintain a stronger association with inattention symptoms than with hyperactivity/impulsivity symptoms.

Moreover, various studies carried out with school-age children have reported moderate relationships between the BRIEF and different rating scales of ADHD symptoms, such as the ADHD rating scale (Mahone et al., 2002) or the BASC (McCandless and O’Laughlin, 2007). In preschool children, even though the research is scarce, significant correlations have been found between the BRIEF indices and Conners’ Parent Rating Scale (Mahone and Hoffman, 2007).

An interesting body of research in the field of education has also shown that, even before children learn to read, the EF can affect the development of essential reading skills. Inhibitory control also has a strong relationship with essential pre-reading skills, such as phonological awareness and letter knowledge (Blair and Razza, 2007), and self-control is related to reading words and pseudowords (Deater-Deckard et al., 2009). Working memory and attentional control have not only been found to have a positive relationship with phonological awareness, but they also predict word and pseudoword reading and recalling the content of a story at the end of preschool (Welsh et al., 2010). The performance on a regulation task, which requires inhibition, attention and working memory, has predictive capacity in the identification of letters and words, and an increase in behavior regulation is associated with better reading scores (McClelland et al., 2007).

Furthermore, some data indicate that working memory evaluations at the age of 5 can predict reading performance years later. A 1-year longitudinal study showed that the EFs of memory, inhibition and mental shifting predicted performance on standardized reading–writing tests, a pattern of results that did not change substantially when fluid intelligence was introduced as the control variable (Neuenschwander et al., 2012). Similarly, Kegel and Bus (2014) conducted a longitudinal study and found, using a fixed-effects analysis to control for time stable confounder, that the change in EFs of working memory and inhibition is related to change in alphabetic skills. Finally, a study conducted with a multilevel mixed-effects model indicated that verbal working memory and dynamic reasoning measures were unique predictors of concurrent and subsequent reading achievement in kindergarten, first and second grade (Stevenson et al., 2014). The relationship between working memory and inhibition and more complex reading skills (e.g., reading comprehension) has also been shown (Demagistri et al., 2014).

The aforementioned findings coincide in highlighting the influential role of the EF in reading acquisition. In the majority of studies so far, performance tests were used, which leads to the question of whether teachers’ EF ratings, which play a fundamental role in the teaching–learning process, can contribute to predicting the development of reading competence.

In summary, in the past decade there has been emphasis on cognitive neuroscience research about the implications of the EF in development (D’Angiulli et al., 2010), and about assessment and intervention procedures, especially in childhood and adolescence. In contrast, very few studies have focused on children in preschool, a period in which the EF make it possible, for example, to listen to and recall instructions, select information, maintain attention on tasks, and manipulate concepts and symbols such as numbers and letters. The EF have traditionally been evaluated with clinical tests in laboratory situations that are not easy to apply in young children (Isquith et al., 2014). Therefore, there is a need for empirical evidence about the agreement between the different EF assessment methods in preschoolers: performance-based tests and rating scales. This could provide relevant information about the validity of the different EF measures and their practical implications. Another topic related to assessment that warrants investigation has to do with the criterion validity of the tests and rating scales, that is, the relationships between these types of instruments and external behavioral and learning criteria.

Taking all these considerations into account, the present study has two different objectives, both related to essential EF components in early childhood:

(1) Study the relationship between parents’ and teachers’ ratings on the BRIEF (Behavioral Regulation Index, Metacognition Index and Working Memory and Inhibition subscales), and preschoolers’ performance on performance-based tests of Verbal Working Memory, Visuospatial Working Memory and Inhibition.
(2) Investigate whether Working Memory and Inhibition performance-based tests, and the BRIEF Meatcognition and Behavioral regulation ratings have any predictive value in relation to indicators of word reading achievement and Inattention and Hyperactivity/impulsivity.

## Materials and Methods

### Participants

The sample was made up of 209 children in the third year of Preschool in the provinces of Castellon and Valencia (Spain). 52.2% of the subjects were boys, and 47.8% girls. 63.3% of the participants came from public schools, 30.6% from government subsidized private schools, and 5.9% from privately owned schools. All the participants received their instruction in the Spanish language. The participants had a mean age of 70.02 months (SD = 3.61; range = 60–76.70 months) and a mean IQ of 98.63 (SD = 12.23; range = 70–126). Children with an IQ of 70 or less were excluded from the sample, based on an equivalence of this measure obtained through the vocabulary and block design subtests (Spreen and Strauss, 1991) of the Wechsler Preschool and Primary Scale of Intelligence (WPPSI; Wechsler, 1967). In addition, subjects with sensorial deficiencies, neurological diseases or serious psychological problems, or those whose families attended social service centers for socio-economic disadvantage, based on information from school records, did not participate in the study. 88.5% of the participants had Spanish nationality, compared to 11.5% who had other nationalities. Regarding the family’s socio-cultural level, 33.5% of the mothers and 39.7% of the fathers had a low educational level (primary school and/or compulsory secondary education), 35.9% of the mothers and 34.9% of the fathers had a medium educational level (high school and/or occupational training), and 30.6% of the mothers and 25.4% of the fathers had higher education studies (university).

### Measures

#### Performance-Based Test of Executive Functioning

##### Inhibition

###### Sun–Moon Stroop task (Archibald and Kerns, 1999)

This task evaluates the subject’s capacity to keep non-pertinent information from interfering in the working memory when carrying out a task, as well as the ability to suppress information that was previously relevant, but that at a certain time is inappropriate. It has two conditions. In condition A, congruent, the subjects are shown a page with 30 images of suns and moons randomly placed in rows and columns. The subjects are told to respond sun to the images with suns and moon to the images with moons as fast as they can (within a period of 45 s). If the child makes a mistake on an image, the experimenter must mark it until the child self-corrects. Right after that, condition B (incongruent) is presented, in which subjects are asked to respond “sun” quickly when the evaluator points to a moon, and “moon” every time the sun is indicated. The task has a high level of reliability, with test–retest scores of 0.91 for the incongruent condition (Archibald and Kerns, 1999). In the present study, the correct trials in the incongruent condition were used.

###### Tapping task (Luria, 1966)

This task requires the subject to learn a pattern of motor responses, maintain this established cognitive attitude, and inhibit the impulse to imitate the examiner’s action (one or two knocks on the table). It also has two conditions, congruent and incongruent, each with 12 stimuli. In the congruent condition, the child has to imitate the experimenter’s action exactly (one or two knocks on the table). In the incongruent condition, when the examiner knocks only one time, the child knocks twice and vice versa. The reliability of the task has been 0.97 (Diamond and Taylor, 1996). The correct trials in the incongruent condition were selected for the analyses.

##### Working memory

###### Digit span (backward; Pickering et al., 1999)

This task consists of 36 items grouped in nine difficulty levels, with each level having one stimulus more than in the previous level (from 2 stimuli on the first level to 8 on the last). The subject must repeat in inverse order a sequence of numbers the experimenter has read aloud. Test–retest reliability of this task is 0.81 (Alloway et al., 2006). The correct trials were used for the analyses.

###### Working memory-counting task (Siegel and Ryan, 1989)

This task has 12 trials grouped in three levels, each with four cards containing randomly placed blue and yellow dots. On each of the levels, the number of cards shown to the subject was increased (two for the first level and four for the last). The child has to count the blue dots on the cards, say this number aloud, and then, after counting the series of cards presented earlier, remember the number of blue dots mentioned in the correct order. Test–retest reliability of this task is 0.62 (Gathercole et al., 2004). The correct trials were used for the analyses.

###### Odd-one-out (Henry and MacLean, 2003)

This task consists of 24 trials grouped in six levels. This task involves showing the child cards containing three similar looking figures in a row from left to right. On each card, two of the figures are identical, with the third differing slightly from the other two. The child is asked to point to the figure that is different from the others – i.e., the odd one out. At each level, the number of cards shown to the subject increases (one for the first level and six cards for the last level). At the end of each trail, the child must recall the location of each different figure in the correct order, pointing with his/her finger to its position (left, center, or right) on a response sheet with blank spaces. Test–retest reliability of this task is 0.81 (Alloway et al., 2006). The correct trials were selected for the analyses.

###### The mazes memory test (Pickering et al., 1999)

This test measures the subject’s ability to recall visuospatial information. The subject is shown a maze with a route drawn on it, and then he/she is asked to trace the same route on a blank maze. The test consists of 12 trials grouped in three levels, each containing four mazes that the child has to fill in according to the model shown by the experimenter. Test–retest reliability of this task is 0.81 (Alloway et al., 2006). The number of correct trials was used for the analyses.

On all the performance-based tests used (both working memory and inhibition tasks), a low score is indicative of deficits in the EF analyzed, as it involves the presence of errors in performing the task.

##### Executive functioning ratings

###### Behavior Rating Inventory of Executive Function (Gioia et al., 2000)

This questionnaire, with versions for parents and teachers, is designed to measure the executive functioning of children and adolescents (5–18 years) in everyday situations through behavioral observation. Its reliability and validity have been widely demonstrated (Clark et al., 2010). In the present study, Cronbach’s alpha was 0.86 for the parent version and 0.99 for teachers. The BRIEF consists of 86 items, scored on a Likert-type scale (1 = Never, 2 = Sometimes, 3 = Often), and grouped in eight scales: inhibit, shift, emotional control, initiate, working memory, plan/organize, organization of materials, and monitor. These scales, in turn, are grouped in two indices: the Behavioral Regulation Index and the Metacognition Index. The sum of the two indices provides a total score on the scale. In this case, high scores indicate risk of executive dysfunction. The present study used the inhibition (e.g., “Is impulsive”) and working memory (e.g., “Cannot stay on the same topic when talking”) scales, as well as indices offered by the questionnaire, given the nature of the performance-based tests applied.

##### ADHD symptoms

###### ADHD criteria

The criteria used were: nine criteria for inattention symptomatology, six for hyperactivity, and the three criteria for impulsivity that appear in the *Diagnostic and Statistical Manual of Mental Disorders* (DSM-IV-TR; American Psychiatric Association, 2000). These were rated by both the parents and teachers of the participants in the study. A dimensional score (from 0 to 2 points) was assigned to these criteria, depending on the frequency of the behavior under observation.

##### Reading achievement

###### Exploration of individual reading difficulties level 1 (Exploración de las Dificultades Individuales de Lectura: Nivel 1, EDIL-1; González, 1987)

The purpose of the test is to analyze abilities and skills from learning objectives in reading instruction. The test is indicated for children with a first reading level, such as students of last year of kindergarten, as stated in the objectives of this educational stage in Spain (see *Ley Orgánica 2/2006, de Educación*). It offers indices of accuracy, speed, reading comprehension, and a global reading level through the different tasks, with an overall test–retest reliability of 0.97 and a criterion validity of 0.71 (González, 1987). For the present study, the word-reading subtest was administered, which consists of 18 words with different difficulty levels placed in a column for the subject to read. The difficulty of each stimulus varies according syllabic structure and length. The accuracy and speed in word reading were taken into account. Both the number of correct answers and the reading time in seconds were used for analyzes.

### Procedure

The research was carried out according to ethical standards. Beforehand, the permission of competent authorities and schools and the parents’ informed consent to perform the study had been obtained. The participants’ assessment took place in classrooms prepared by the different schools to meet all the necessary conditions for applying the psychological tests. The tests were administered by two professionals and a research technician, a doctoral student in Cognitive Neuroscience and Education with experience in the application and scoring norms. Two sessions were used for each child, each one lasting approximately 30 min. The order in which the tests were administered to the children in each of the two sessions was random across successive tests, so that fatigue would not introduce biases in the results. All the tests and instructions during the assessment were administered in the Spanish language. Children received stickers for participating.

The parents’ and teachers’ rating scales were given to the recipients in sealed envelopes, and they were returned to the experimenters to check that they were filled out properly. As compensation, the families and schools were given a report about the child’s performance in the different domains analyzed.

## Results

The data analyses were conducted with the SPSS statistical package, version 19.00. First, descriptive analysis of the different tests used were performed (see **Table 1**).The relationship between the performance-based tests and the executive functioning rating scales was analyzed using bivariate correlation analysis (Pearson). To examine the predictive power of the performance-based test on ADHD behaviors (inattention and hyperactivity/impulsivity) and on reading achievement (word reading accuracy and speed), multiple linear regression analysis was performed using the stepwise method. Regression analyses were also carried out using the same procedure to analyze the predictive power of the parents’ and teachers’ executive functioning ratings from the BRIEF on ADHD symptoms. Finally, multiple regression analyses were carried out with the parents’ and teachers’ EF ratings from the BRIEF on the participants’ reading achievement.

**Table 1:** Descriptive analysis.

	Mean	SD	Range
**EF performance-based test**
Stroop	33.01	6.79	14–56
Tapping	10.71	1.78	3–12
Digits	5.28	2.48	0–16
Counting	5.67	2.53	0–12
Odd-one-Out	6.10	2.36	1–15
Mazes	3.85	2.30	0–11
**EF rating scales**
*Teacher’s version*			
Inhibition	13.69	4.75	10–30
Working Memory	14.87	5.30	10–30
Behavioral Regulation Index	39.07	11.785	29–81
Matcognition Index	63.41	19.32	44–123
*Parent’s Version*			
Inhibition	15.62	3.62	10–28
Working Memory	15.01	3.79	10–28
Behavioral Regulation Index	43.76	8.54	28–71
Matacognition Index	69.34	13.38	44–11
**ADHD symptoms**
*Teacher’s report*			
Inattention	15.62	3.62	0–18
Hyperactivity/Impulsivity	15.01	3.79	0–18
*Parent’s report*			
Inattention	3.99	3.26	0–13
Hyperactivity/Impulsivity	4.87	3.86	0–17
**Word-reading**
Correct answers	13.48	6.14	0–18
Time (seconds)	74.31	52.70	20–300

As **Table 2** shows, all statistically significant correlations were negative, given the opposite direction of the scores of the executive functioning measures used. The teachers’ ratings on the BRIEF Index and subscales (Metacognition Index, Behavioral Regulation Index, Inhibition and Working Memory) correlated significantly with all the performance-based tests of Inhibition and Verbal Working Memory. The correlation values between the Working Memory ratings and the Stroop Sun/Moon Inhibition test, *r*(209) = -0.40, *p* < 0.001, and with the verbal working memory counting task, *r*(209) = -0.46, *p* < 0.001, were moderate. Moreover, the Metacognition Index from the BRIEF had a moderate correlation with these same tests: Stroop Sun/Moon, *r*(209) = -0.35, *p* < 0.001, and Counting, *r*(209) = -0.43, *p* < 0.001.

**Table 2:** Correlation among EF measures: performance-based tests and teacher and parent ratings (*N* = 209).

	Inhibition		Verbal WM		Visuospatial WM	
	Stroop	Tapping	Digits	Counting	Odd-one-out	Mazes
**BRIEF teachers’ version**
Inhibition	-0.209^∗∗^	-0.156^∗^	-0.165^∗^	-0.178^∗^	–	-0.150^∗^
Working Memory	-0.402^∗∗^	-0.274^∗∗^	-0.279^∗∗^	-0.456^∗∗^	-0.257^∗∗^	-0.317^∗∗^
Behavioral Reg. Index	-0.203^∗∗^	-0.141^∗^	-0.184^∗∗^	-0.208^∗∗^	–	-0.151^∗^
Metacognition Index	-0.350^∗∗^	-0.247^∗∗^	-0.270^∗∗^	-0.429^∗∗^	-0.226^∗∗^	-0.301^∗∗^
**BRIEF parents’ version**
Inhibition	–	–	–	–	–	-0.179^∗∗^
Working Memory	-0.350^∗∗^	-0.267^∗∗^	-0.245^∗∗^	-0.417^∗∗^	-0.297^∗∗^	-0.294^∗∗^
Behavioral Reg. Index	–	–	–	–	–	-0.167^∗^
Metacognition Index	-0.286^∗∗^	-0.248^∗∗^	-0.243^∗∗^	-0.379^∗∗^	-0.270^∗∗^	-0.271^∗∗^

^∗^*p* < 0.05; ^∗∗^*p* < 0.001. Reg., Regulation.

Likewise, the Mazes Visuospatial Working Memory test had statistically significant relationships with all the teachers’ EF ratings on the BRIEF, specifically, Working Memory, *r*(209) = -0.32, *p* < 0.001) and Metacognition Index, *r*(209) = -0.30, *p* < 0.001. The Visuospatial Memory test “Odd-one-out” also correlated significantly with the teachers’ EF ratings, except Inhibition and the Behavioral Regulation Index.

On the other hand, Working Memory and the Metacognition Index from the BRIEF rated by the parents had statistically significant correlations with the performance-based tests of Inhibition and Verbal and Visuospatial Working Memory. Especially noteworthy are the correlations between the BRIEF Working Memory scale and the Stroop Sun/Moon test, *r*(209) = -0.35, *p* < 0.001, and the Verbal Memory Counting test, *r*(209) = -0.42, *p* < 0.001, and between the Metacognition Index and the Counting test, *r*(209) = -0.38, *p* < 0.001). The Inhibition Scale and the Behavioral Regulation Index only correlated significantly with the Mazes Visuospatial Memory test.

The performance-based test of Counting (ΔR^2^ = 0.224; *p* < 0.001), Stroop Sun/Moon (ΔR^2^ = 0.057; *p* < 0.001) and Mazes (ΔR^2^ = 0.024; *p* = 0.008) tests together predicted 30.5% of the Inattention symptoms, while only 7.6% of Hyperactivity/impulsivity was predicted by the Counting (ΔR^2^ = 0.057; *p* < 0.001) and Stroop Sun/Moon (ΔR^2^ = 0.037; *p* = 0.042) tests (see **Table 3**).

**Table 3:** Multiple linear regression analysis for performance-based EF tests predicting ADHD behaviors and reading performance.

	*F*	R2	ΔR2	Beta
**DSM-IV: Inattention**
Counting	59.91^∗∗^	0.224	0.224	-0.319
Stroop	40.30^∗∗^	0.281	0.057	-0.250
Mazes	30.01^∗∗^	0.305	0.024	-0.165
**DSM-IV: Hyperactivity/Impulsivity**
Counting	12.57^∗∗^	0.057	0.057	-0.180
Stroop	8.48^∗∗^	0.076	0.019	-0.150
**Reading: Word accuracy**
Counting	74.21^∗∗^	0.264	0.264	0.389
Stroop	42.50^∗∗^	0.292	0.028	0.155
Digits	30.39^∗∗^	0.308	0.016	0.143
**Reading: Time on words**
Stroop	28.24^∗∗^	0.136	0.136	-0.296
Digits	20.75^∗∗^	0.189	0.053	-0.241

*^∗^p* < 0.05; *^∗∗^p* < 0.001.

On the reading achievement indicators, the performance-based test of Counting (ΔR^2^ = 0.264; *p* < 0.001), Stroop Sun/Moon (ΔR^2^ = 0.028; *p* = 0.005) and Digits Memory (ΔR^2^ = 0.016; *p* = 0.032) tests predicted 30.8% of word reading accuracy. Regarding reading time, the Stroop Sun/Moon (ΔR^2^ = 0.136; *p* < 0.001) and Digits (ΔR^2^ = 0.053; *p* = 0.001) tests predicted 18.9% of the variance.

Regarding the ADHD symptoms, the BRIEF Metacognition Index rated by the teacher (ΔR^2^ = 0.761; *p* < 0.001) explained 76.1% of the variance in the scores on Inattention. Likewise, the parents’ ratings on the Metacognition Index (ΔR^2^ = 0.520; *p* < 0.001) explained 52% of the variance in inattention behaviors. 53.3% of the total variance of Hyperactivity/Impulsivity was predicted by the teachers’ ratings on the Behavioral Regulation Index (ΔR^2^ = 0.533; *p* < 0.001), while in the case of the parents’ version, 33.6% of hyperactivity/impulsivity was predicted by the Behavioral Regulation Index (ΔR^2^ = 0.320; *p* < 0.001) and the Metacognition Index (ΔR^2^ = 0.016; *p*= 0.026; see **Table 4**).

**Table 4:** Regression analysis of the EF rating scales (parents’ and teachers’ versions) on ADHD symptoms and reading performance measures.

	BRIEF teachers’ version	BRIEF parents’ version			
	*F*	R2	ΔR2	Beta	*F*	R2	ΔR2	Beta
**DSM-IV: Inattention**
Metacognition Index	660.53^∗∗^	0.761	0.761	0.873	223.47^∗∗^	0.520	0.520	0.721
Behavioral Reg. Index	n.s.				n.s.			
**DSM-IV: Hyperactivity/Impulsivity**
Behavioral Reg. Index	235.86^∗∗^	0.533	0.533	0.730	97.07^∗∗^	0.320	0.320	0.480
Metacognition Index	n.s.				51.98^∗∗^	0.336	0.016	0.154
**Reading: Word accuracy**
Metacognition Index	68.60^∗∗^	0.249	0.249	-0.627	28.47^∗∗^	0.121	0.121	-0.348
Behavioral Reg. Index	38.86^∗∗^	0.264	0.015	0.176	n.s.			
**Reading: Time on words**
Metacognition Index	25.31^∗∗^	0.165	0.165	0.406	22.96^∗∗^	0.114	0.114	-0.338
Behavioral Reg. Index	n.s.				n.s.			

^∗^*p* < 0.05; ^∗∗^*p* < 0.01. Reg., Regulation.

On the reading achievement measures, the teachers’ versions of the Metacognition Index (ΔR^2^ = 0.249; *p* < 0.001) and the Behavioral Regulation Index (ΔR^2^ = 0.015; *p* < 0.001) together predicted 26.4% of word reading accuracy. Regarding the parents, the Metacognition Index (ΔR^2^ = 0.121; *p*= 0.045) only predicted 12.1% of the variance in word reading accuracy. The teachers’ ratings on the Metacognition Index (ΔR^2^ = 0.165; *p* < 0.001) predicted 16.5% of word reading time, while parents’ ratings predicted 11.4% (ΔR^2^ = 0.114; *p* < 0.001).

## Discussion

The assessment of emergent forms of EF is useful for early identification and intervention in neurodevelopmental disorders, especially ADHD, autism spectrum disorders and specific learning disabilities, which involve an early executive dysfunction (Meltzer, 2007). However, research on procedures for measuring the executive skills in the preschool period is still scarce. Thus, the first objective of the present study is to analyze the relationship between two different EF assessment methods in preschoolers, parents’ and teachers’ BRIEF ratings and classic performance-based tests of inhibition and working memory.

Coinciding with recent studies (Alloway et al., 2009; Toplak et al., 2009), the values of the correlations between the two procedures were only moderate, although they were somewhat higher than those found in other studies (Mahone and Hoffman, 2007; McAuley et al., 2010). It is plausible to suppose that, although they share a neuroanatomical substrate (Mahone et al., 2009), the two types of measures assess different aspects of the same construct, so that the BRIEF seems to measure essentially the behavioral component of the EF, while the performance-based tests are more focused on evaluating the cognitive component (Anderson et al., 2002).

However, it is important to highlight the variability observed in the agreement between the assessment methods depending on the different executive processes being evaluated. Thus, Working Memory and the Metacognition Index from the BRIEF, rated by both the parents and the teachers, presented important correlations with the Verbal Working Memory Counting test, and an identical pattern of associations occurs with the Digits Verbal Working Memory and the Mazes Visuospatial Memory test, although with lower values. This panorama as a whole shows that the observation of everyday activities that require the application of memory skills has an appreciable connection with the performance on tests in a structured context. Identifying the profile of strengths and weaknesses in Working Memory in different situations and activities is quite useful in designing supports. Moreover, the parents’ and teachers’ Working Memory ratings show significant correlations with the Stroop Sun/Moon test. Another interesting finding from our study, coinciding with a previous study (Parrish et al., 2007), is the important association between the Stroop Sun/Moon Inhibition test and the Metacognition Index on the BRIEF.

Moreover, regarding the informants’ ratings, it is surprising that the correlations between the parents’ ratings of Inhibition and Behavioral Regulation Index and the performance-based tests did not reach statistical significance, not even in the case of the inhibition tests, with which they seem to share a conceptual framework. However, in the case of the teachers, coherently, the associations between the aforementioned variables are clear. The differences in terms of the informant (parents versus teachers) could be explained by the fact that parents of children of these ages have an emotional involvement bias toward permissiveness about poorly regulated behaviors. It is also possible that teachers are more competent evaluators because they have more experience with developmental normative references than parents do, or that parents and teachers have different expectations about development (Wolraich et al., 2004).

Regarding the second objective, our findings about the predictive power of the two types of measures on the core ADHD symptoms show that three performance-based tests, Verbal Working Memory, Visuospatial Working Memory and Inhibition, explained 30.5% of the Inattention symptoms. These results show the existing relationship between the ADHD symptomatology and the performance on performance-based executive functioning tests from early developmental periods (Sonuga-Barke et al., 2003; Thorell and Wåhlstedt, 2006; Sims and Lonigan, 2012; Schoemaker et al., 2013). Moreover, in our sample the ADHD symptoms were not only related to the component of inhibition, but working memory also played an important role. The predictive capacity of the BRIEF indices was superior to that of the performance-based tests, as the teachers’ ratings were able to explain 76.1% of the variance in inattention, and the parents’ ratings explained 52%. Although with lower values, the prediction of Hyperactivity/impulsivity behaviors painted a similar picture: a relatively low predictive value of the Counting and Stroop Sun/Moon performance-based tests, and a moderate value of the parents’ BRIEF ratings, which was higher in the case of the teachers’ ratings. In general terms, the results confirm that EF deficits are more related to inattention symptoms than to those of hyperactivity/impulsivity (Brocki et al., 2010; Willcutt et al., 2012). More specifically, coinciding with previous contributions, the typical disassociation was observed between the BRIEF indices and the ADHD symptoms; that is, the Metacognition Index presented a greater relationship than the Behavioral Regulation Index with inattention symptoms, while the Behavioral Regulation Index presented a greater relationship with symptoms of hyperactivity/impulsivity (Mahone et al., 2002; McCandless and O’Laughlin, 2007; McAuley et al., 2010).

The percentages of variance of the two reading performance indicators, accuracy and time on reading words, were quite different, low in the case of time and acceptable in the case of word reading. Specifically, the two verbal working memory (Counting and Digits) performance-based tests, together with the Stroop Sun/Moon, showed a noteworthy predictive power that was slightly higher than that of the teachers’ BRIEF indices. One interesting conclusion from a longitudinal study related to our results was that better performance on remembering digits and executive skills provided the children with an immediate head start in math and reading, which they maintained throughout the first 3 years of primary school (Bull et al., 2008). Such kind of findings are highlighting the important role of EF tests in predicting academic skills (Welsh et al., 2010; Miranda et al., 2012; Neuenschwander et al., 2012; Richland and Burchinal, 2013). However, there might be functional dependency between reading and EF. Reading learning may improve early executive functioning, as it has been demonstrated in narrative skills or social interaction domains (Friend and Bates, 2014; Moriguchi, 2014).

### Limitations and Strengths

As in making decisions about treatments, the selection of performance-based assessment tests has to be based on advances supported by scientific evidence. One of the strengths of the present study is that, to the best of our knowledge, it makes a pioneering attempt to empirically evaluate the relationship between two EF assessment methods in preschoolers, and determine their predictive power on two different functional criteria: ADHD symptoms and word reading performance. Even though this is an essential issue for validating these measures, the scant literature has been restricted to studies with adults that found EF ratings to be significantly related to impairments in life activities and job-related functioning (Barkley and Fischer, 2011).

The present study reports that, in preschoolers, agreement between the two types of methodologies, EF ratings and performance tests, is only moderate, suggesting that they are assessing different aspects of cognitive and behavioral functioning. However, both measurement procedures present a significant relationship with fundamental external criteria for adapting to school, such as reading and inattention and hyperactivity/impulsivity behaviors, which shows their practical value. Another strength of the present study is that it shows the role teachers play in rating the executive skills of young children. Although studies have usually used information from parents, the present study highlights that teachers’ ratings present even higher relationships than those of the parents, with both the performance-based tests and the ADHD symptom and reading achievement variables.

The contribution of this study has to be evaluated taking into account a series of limitations. Although numerous, the sample used was made up of 5-years-old children, so that the results cannot be generalized to other ages or periods of development. It would be advisable, therefore, to replicate the findings, broadening the age range of the sample at least to 3 and 4-years-old children, in order to address the whole period of preschool education. Other limitations are related to the battery of performance-based tests selected. Even though various tasks were applied to assess the essential EFs, working memory and inhibition, no measures were included to assess other important components, such as cognitive flexibility or planning. Moreover, the present study has only referred to the cool EF skill components, so that future studies will have to broaden the perspective to include emotional regulation, which can be considered a behavioral manifestation of hot EF skill components.

### Practical Significance

Executive function ratings add a complementary tool to performance-based test that measure more specific components. While performance-based tests seem to be better at predicting reading performance, behavioral ratings show a greater capacity to predict ADHD symptoms. As Toplak et al. (2013, p. 131) conclude in an excellent review on the topic: “These two types of measures appear to capture different levels of cognition, namely, the efficiency of cognitive abilities and success in goal pursuit.” Thus, the combined application of both types of measures would provide a more comprehensive description of executive deficits related to learning processes and adverse behavioral manifestations.

Given their relative ease of use in terms of speed and economy of information from parents and teachers, questionnaires like the BRIEF are valuable instruments for screening possible disorders in development, behavior and learning in the preschool period. Information from multiple contexts would provide a more complete and ecological profile of the strengths and limitations of each child and, consequently, make it possible to design more effective interventions at home and at school.

Recently, intervention programs framed in the neurocognitive perspective of EF have been created. Although exploratory in nature, they show great possibilities. A prototypical example is the program “Tools of the Mind,” elaborated by Diamond and colleagues for preschoolers, and based on the influence of physical, social and emotional experiences in the prefrontal cortex (Diamond, 2010; Diamond and Lee, 2011). Computer training in neurocognitive processes, especially executive attention, is a less ecological approach, but it has also been shown to foster the development of the processes being trained (see review by Rueda et al., 2010). Furthermore, the data show that not only executive functioning improves with ad hoc training, but improvements are also observed on neurophysiological measures. Specifically, there is evidence that changes produced by training young children (4 years-old) to think about their representational rules are associated with a reduction in the amplitude of the N2 component of the event-related potentials (Espinet et al., 2013).

## Conflict of Interest Statement

The authors declare that the research was conducted in the absence of any commercial or financial relationships that could be construed as a potential conflict of interest.

## Acknowledgments

This work is supported by the Ministry of Economy and Competitiveness of Spain (EDU2012-37452) and the University of Castellon Jaume I (pre-doctoral fellowship 2I005-PREDOC/2013/34).
